# Diagnostic Utility of PRAME and SOX10 Immunostaining for Vulvar Melanocytic Lesions

**DOI:** 10.7759/cureus.103309

**Published:** 2026-02-09

**Authors:** Kridtin Jarutatsanangkoon, Phitsinee Purngpiputtrakul, Suchanan Hanamornroongruang, Vuthinun Achariyapota, Panitta Sitthinamsuwan

**Affiliations:** 1 Pathology, Faculty of Medicine, Siriraj Hospital, Mahidol University, Bangkok, THA; 2 Dermatology, Faculty of Medicine, Siriraj Hospital, Mahidol University, Bangkok, THA; 3 Obstetrics and Gynecology, Faculty of Medicine, Siriraj Hospital, Mahidol University, Bangkok, THA

**Keywords:** diagnosis, genital lentigine, genital melanocytic nevus, immunostaining, mucosal lentiginous melanoma, prame, preferentially translated antigen in melanoma, sox10, special site nevus, vulvar melanoma

## Abstract

Background: Vulvar melanoma (VM) is a rare type of malignant melanoma. Clinical presentations typically show mass or bleeding. Benign melanocytic lesions of the vulva include genital lentigines and genital melanocytic nevus. SOX10 is a marker commonly used in melanocytic lesions, and the preferentially expressed antigen in melanoma (PRAME) is a recent marker that shows high specificity for malignant melanoma.

Objectives: The study is to evaluate the histopathology and immunohistochemistry of SOX10 and PRAME in VM and benign vulvar melanocytic lesions (genital lentigine and genital melanocytic nevus).

Materials and methods: All cases of VM and benign vulvar melanocytic lesions were recruited. Clinical and pathological data were reviewed. SOX10 and PRAME immunohistochemistry were performed and compared between VM and benign vulvar melanocytic lesions.

Results: Of the total nine cases with VM, 6 (66.7%) presented with mass, 2 (22.2%) had mass with bleeding, and one (11.1%) developed urinary discomfort. Histopathologically, mucosal lentiginous melanoma is the most common subtype. Thirteen cases with benign melanocytic lesions were found. In immunohistochemical studies, SOX10 showed positivity in all 9 (100%) VM and all 13 (100%) benign melanocytic lesions. PRAME showed positivity in 7 (77.8%) cases. All 13 (100%) benign melanocytic lesions show negative stains for PRAME. The sensitivity of SOX10 and PRAME in the diagnosis of VM was 100% and 88.9%, respectively. The specificity of SOX10 and PRAME in the diagnosis of VM was 0% and 100%, respectively.

Conclusions: SOX10 and PRAME are nuclear staining markers that can be used for diagnostic purposes and as adjunct markers for the diagnosis of VM and benign melanocytic lesions. PRAME immunostaining shows high specificity for melanoma and melanoma in situ.

## Introduction

Malignant melanoma of the female genital tract is a rare condition that accounts for 1% of all malignancies of the female genital tract and 3% of all melanomas [1,2]. The mean age of patients with vulvar melanoma (VM) is in the sixth to seventh decade, and the most common presenting symptoms are abnormal bleeding (34.8%) and mass (28.3%) [2-5]. The lesions typically appear as hyperpigmented lesions with or without ulceration and microsatellites, although amelanotic lesions can also occur [3,6]. The predominant histological subtypes are nodular, mucosal lentiginous, and superficial spreading. Neoplastic cells exhibit various morphological features, such as epithelioid, spindle, or other rare variants, such as pseudoglandular pattern, rhabdoid feature, and small cell type [6,7]. The main treatment modality is surgical resection, with adjuvant radiotherapy in some cases. The prognosis of patients with VM is generally poor, with a median survival of 53 months [8-11].

Genital melanosis (GM) is a hyperpigmented lesion in the vulva that has been reported to be associated with VM. Clinically, GM revealed irregular hyperpigmented patches, which mimic melanoma. GM can be histologically classified into two types: Genital lentigines (GL) and genital melanotic macules. GL exhibits melanocytic proliferations, whereas the genital melanotic macule shows hyperpigmentation of the basal layer without melanocytic proliferation. The relationship between GL and VM is unclear and controversial. The pathogenesis of VM and its possible link to GL are still under investigation [12]. Histologically, GL is difficult to distinguish from melanoma in situ when it is present in the background of VM. Diagnosis and margin assessment of VM with a background of GL were challenging.

Benign melanocytic nevi in the genital area are classified as common melanocytic nevi, congenital melanocytic nevi, and special site nevi. By clinical and histopathological features, common and congenital melanocytic nevi can be clearly distinguished from VM. On the other hand, special site nevi usually present with a larger size, irregular borders, and discoloration. Furthermore, its atypical histopathological features can mimic melanoma [13]. These atypical findings consist of an asymmetrical lesion with architectural atypia, an irregular junctional lentiginous proliferation with irregular nest formation, pagetoid spreading, and cellular atypia, including increased sized of nuclei of melanocytes and dermal fibrosis.

VM is typically positive for immunostaining markers such as S100, HMB45, and Melan A and negative for non-melanocytic markers. Another sensitive and specific marker for melanocytic lesions is SOX10 immunohistochemistry. This marker shows nuclear positivity in almost all primary and metastatic melanoma and melanocytic nevi. SOX10 can be useful to identify melanoma with unusual features and to assess the melanocytic proliferation in genital melanosis [14-16].

Preferentially expressed antigen in melanoma (PRAME), a novel immunohistochemical marker, is predominantly expressed in the nucleus of melanoma cells and some other neoplastic cells [17,18]. PRAME immunohistochemistry is useful for differentiating melanoma from benign melanocytic lesions and can also be used in conjunction with other markers such as Melan-A and HMB-45 to enhance the accuracy and sensitivity of melanoma diagnosis [18-20].

The aim of this study is to investigate histopathological characteristics and subtypes of VM and the comparison of SOX10 and PRAME expression between VM and benign vulvar melanocytic lesions.

## Materials and methods

All cases of VM, melanoma in situ, and benign melanocytic lesions (including GL and genital melanocytic nevus) diagnosed at the Department of Pathology, Faculty of Medicine, Siriraj Hospital from January 2006 to July 2022 were recruited. All paraffin blocks have been retrieved for the study. Metastatic melanoma from other sites was excluded.

Demographic data, including age at diagnosis, underlying disease, clinical manifestation, physical examination, pathological staging, operation, and records of radiotherapy and chemotherapy, were collected. Benign melanocytic lesions were recruited as a control. These benign lesions included melanocytic nevi and GL.

All slides of VM and benign melanocytic lesions were reviewed by an experienced dermatopathologist (PS), a general pathologist (KJ), and a trainee in pathology (PP). The diagnostic criteria are based on the World Health Organization (WHO) Classification of Skin Tumours [21]. The following data, including site, size, Breslow thickness, vertical growth and radial growth, histologic type, ulceration, microsatellite, Clark’s level, mitotic rate, vascular and perineural invasions, tumor infiltrating lymphocytes, tumor regression, resection margin, nodal status data, and extranodal extension, were recorded for VM. Melanoma in situ in VM cases was recorded.

Paraffin blocks were selected for immunohistochemical (IHC) stain. All specimens were cut into 3-4 μm-thick slices to make sections for IHC stain using a fully automated assay for SOX10 (BioCare Medical® SOX10 (M) (BC34) mouse monoclonal antibody) and PRAME (Quartett Anti-PRAME (Clone QR005) rabbit monoclonal antibody). SOX10 was interpreted as either a positive or negative stain. The staining data of PRAME were classified by the percentage of immunoreactive melanocytes, including negative stain (0%), positive stain in 1-25%, 26-50%, 51-75%, and 76-100% of tumor cells, and intensity of IHC stain, including negative stain, weak staining, moderate staining, and strong staining in VM, melanoma in situ, and benign melanocytic lesions. PRAME was evaluated as positive staining when present with nuclear staining in >75% of the neoplastic cells.

The protocol for this study was ethically approved by the Siriraj Institutional Review Board (SIRB) (Certificate of approval number Si 655/2022). This study did not require written informed consent to participate. The current article did not reveal any personally identifiable information about research participants. Written informed consent was not required for publishing. All data in the study were kept confidentially in accordance with the Declaration of Helsinki.

Statistical analysis in this study was performed using SPSS Statistics, v. 18.0 (SPSS Inc., Chicago, Illinois). The clinical information and pathological findings were analyzed with descriptive statistics. The chi-square or Fisher’s exact test was utilized in the comparison between PRAME and SOX10 immunostaining to differentiate VM, melanoma in situ, and benign melanocytic lesions. Sensitivity, specificity, positive predictive value, negative predictive value, positive likelihood ratio, negative likelihood ratio, and accuracy were calculated.

## Results

In total, nine (100%) VM cases were enrolled. No case with melanoma in situ alone was observed. The average age of the melanoma patients was 67 years (range 32-87). The clinical presentation of 9 (100%) patients with VM included a mass in 6 (66.7%) patients, a mass with bleeding in 2 (22.2%), and urinary discomfort in 1 (11.1%). Physical examination showed single lesions in 8 (88.9%) and multiple lesions in 1 (11.1%). Melanoma in situ was encountered in all cases of VM. Demographic data and treatment of the patients are detailed in Table 1. Of 13 (100%) cases with benign melanocytic lesions, they comprised 5 (38.5%) GLs, 4 (30.8%) congenital melanocytic nevi, 3 (23.1%) common intradermal melanocytic nevi, and 1 (7.6%) special site nevus. All 5 (100%) GLs were found to be associated with VM.

**Table 1:** Demographic data and treatment of vulvar melanoma patients (total n = 9)

Clinical characteristics		Number of cases (%)
Age in years (range)	Mean 67 (range 32–87)	
Chief complaints	Mass	6 (66.7)
	Mass with bleeding	2 (22.2)
	Urinary discomfort	1 (11.1)
Tumor focality	Single	8 (88.9)
	Multiple	1 (11.1)
Pathological tumor (pT) staging	1a	0 (0)
	1b	0 (0)
	2a	1 (11.1)
	2b	2 (22.2)
	3a	0 (0)
	3b	2 (22.2)
	4a	0 (0)
	4b	4 (44.4)
Procedure	Biopsy	3 (33.3)
	Excision/wide excision	3 (33.3)
	Vulvectomy	3 (33.3)
Chemotherapy	Yes	0 (23.1)
	No	9 (76.9)
Radiation therapy	Yes	5 (55.6)
	No	4 (44.4)
Node dissection	Yes	4 (44.4)
	No	5 (55.6)

**Figure 1:**
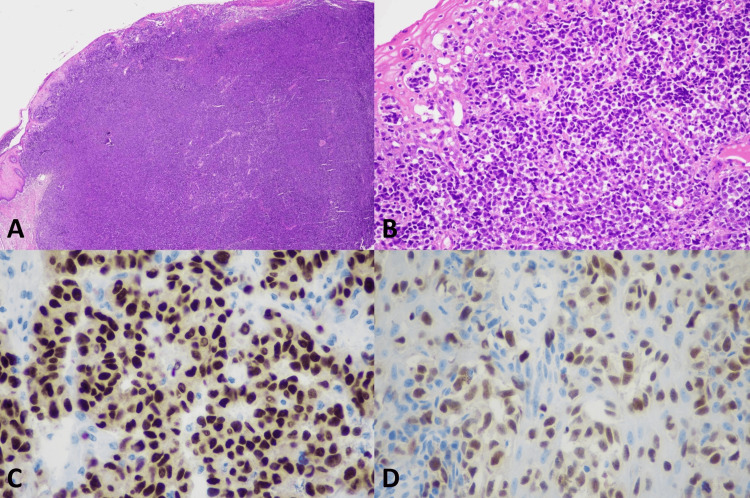
Histopathology of mucosal lentiginous melanoma (A) Vulvar melanoma, mucosal lentiginous melanoma (original magnification ×20), (B) Overlying mucosa showing pagetoid spreading (original magnification ×200), (C) Positive nuclear staining of SOX10 (original magnification ×400), (D) Positive nuclear staining of PRAME (original magnification ×400)

**Figure 2:**
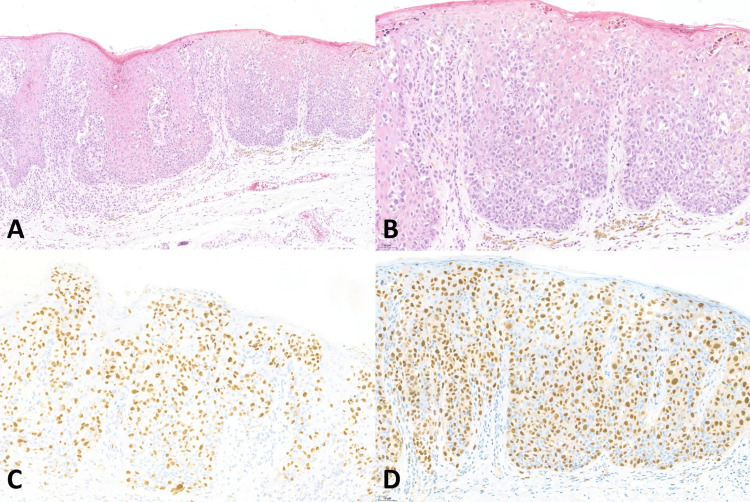
Histopathology of superficial spreading melanoma (A) Vulvar melanoma, superficial spreading melanoma (original magnification ×100), (B) Overlying mucosa showing pagetoid spreading (original magnification ×200), (C) Positive nuclear staining of SOX10 (original magnification ×200), (D) Positive nuclear staining of PRAME (original magnification ×200)

Histopathology findings in 9 (100%) VM patients are shown in Table 2. Identified morphological features were epithelioid cell features in 5 (55.6%), spindle cell features in 2 (22.2%), small blue round cell features in 1 (11.1%), and pseudoglandular features in 1 (11.1%). The types identified were mucosal lentiginous melanoma in 7 (77.8%) (Figure 1) and superficial spreading melanoma in 2 (22.2%) (Figure 2). Breslow thickness was 1.5-28 mm in depth and 4 mm in median. Clark levels of the tumor were level III in 3 (33.3%), level IV in 5 (55.5%), and level V in 1 (11.1%). Partial tumor regression was present in 5 (55.6%) cases. Lymphovascular invasion was present in 3 (33.3%) cases, and perineural invasion was identified in 2 (22.2%) cases. Melanoma in situ was encountered in all 9 VM patients (100%).

**Table 2:** Pathological findings of vulvar melanoma cases (total n = 9) Abbreviations: n, number; SD, standard deviation

Pathological characteristics		Results
Tumor laterality, n (%)	Right	4 (44.4)
	Left	3 (33.3)
	Central	2 (22.2)
Tumor size in cm, mean ± SD (range)		3.6 ± 2.2 (0.3–9)
Histology subtype, n (%)	Mucosal lentiginous type	7 (77.8)
	Superficial spreading type	2 (2.2)
Tumor cell type, n (%)	Epithelioid	5 (55.5)
	Spindle	2 (2.2)
	Small blue round cell	1 (11.1)
	Pseudoglandular	1 (11.1)
Breslow thickness (mm), median (range)		4 (1.3–28)
Ulceration, n (%)	Present	8 (88.9)
	Not identified	1 (11.1)
Microsatellite, n (%)	Present	1 (11.1)
	Not identified	8 (88.9)
Clark level, n (%)	1	0 (0)
	2	0 (0)
	3	3 (33.3)
	4	5 (55.5)
	5	1 (11.1)
Lymphovascular invasion, n (%)	Present	3 (33.3)
	Not identified	6 (66.6)
Perineural invasion, n (%)	Present	2 (22.2)
	Not identified	7 (77.8)
Tumor infiltrating lymphocytes (TIL), n (%)	None	4 (44.4)
	Present, non-brisk	5 (55.5)
	Present, brisk	0 (0)
Tumor regression, n (%)	Present	5 (55.5)
	Not identified	4 (44.4)
Genital lentigines, n (%)	Present	5 (55.5)
	Not identified	4 (44.4)
Melanoma in situ, n (%)	Present	9 (100)
	Not identified	0 (0)

**Figure 3:**
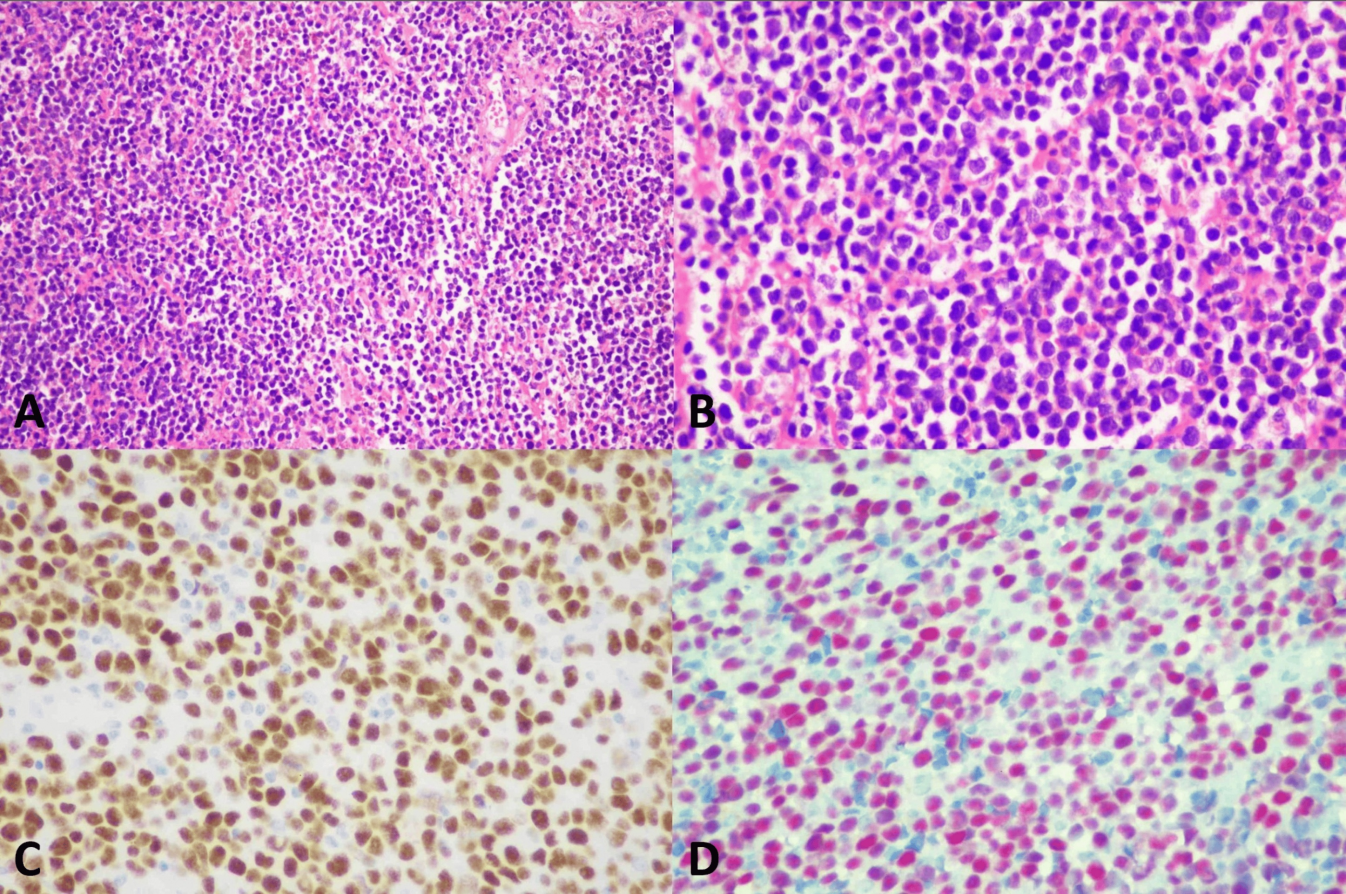
Histopathology of melanoma with small cell morphology (A) Vulvar melanoma (original magnification ×100), (B) Small cell morphology (original magnification ×200), (C) Positive nuclear staining of SOX10 (original magnification ×200), (D) Positive nuclear staining of PRAME (original magnification ×200)

**Figure 4:**
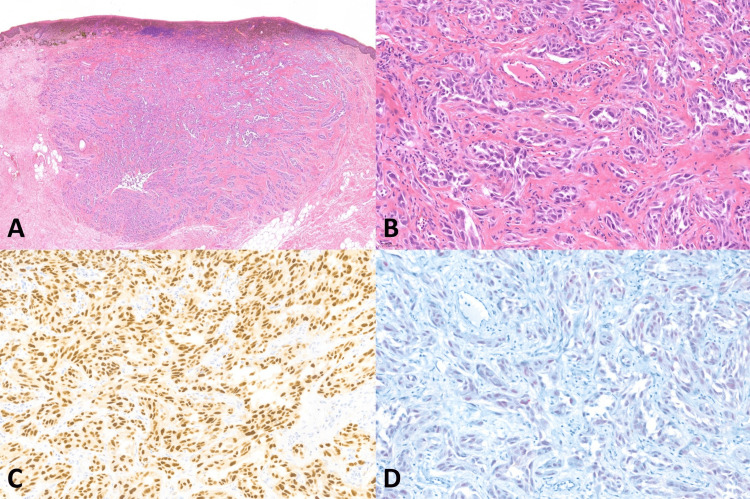
Histopathology of melanoma with pseudoglandular morphology (A) Vulvar melanoma (original magnification ×20), (B) Pseudoglandular morphology (original magnification ×200), (C) Positive nuclear staining of SOX10 (original magnification ×200), (D) Positive nuclear staining of PRAME (original magnification ×200)

**Figure 5:**
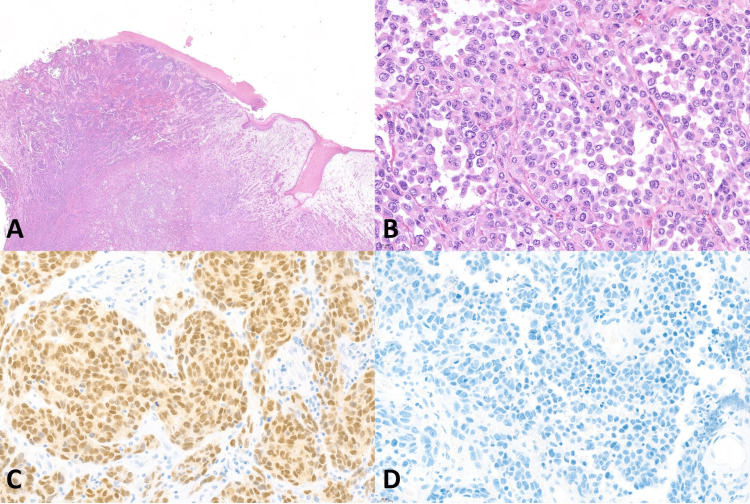
Histopathology of melanoma with epithelioid morphology (A) Vulvar melanoma (original magnification ×20), (B) Epithelioid morphology (original magnification ×400), (C) Positive nuclear staining of SOX10 (original magnification ×400), (D) Negative nuclear staining of PRAME (original magnification ×400)

**Figure 6:**
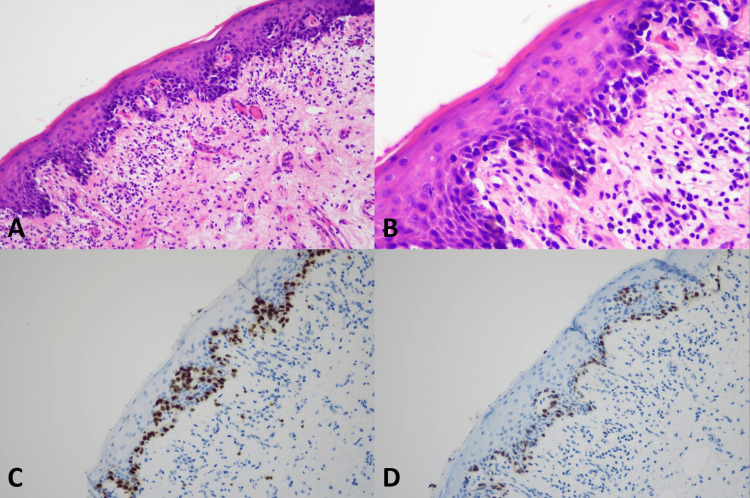
Histopathology of melanoma in situ (A) Vulvar melanoma in situ (original magnification ×100), (B) Melanoma in situ (original magnification ×200), (C) Positive nuclear staining of SOX10 (original magnification ×200), (D) Positive nuclear staining of PRAME (original magnification ×200)

**Figure 7:**
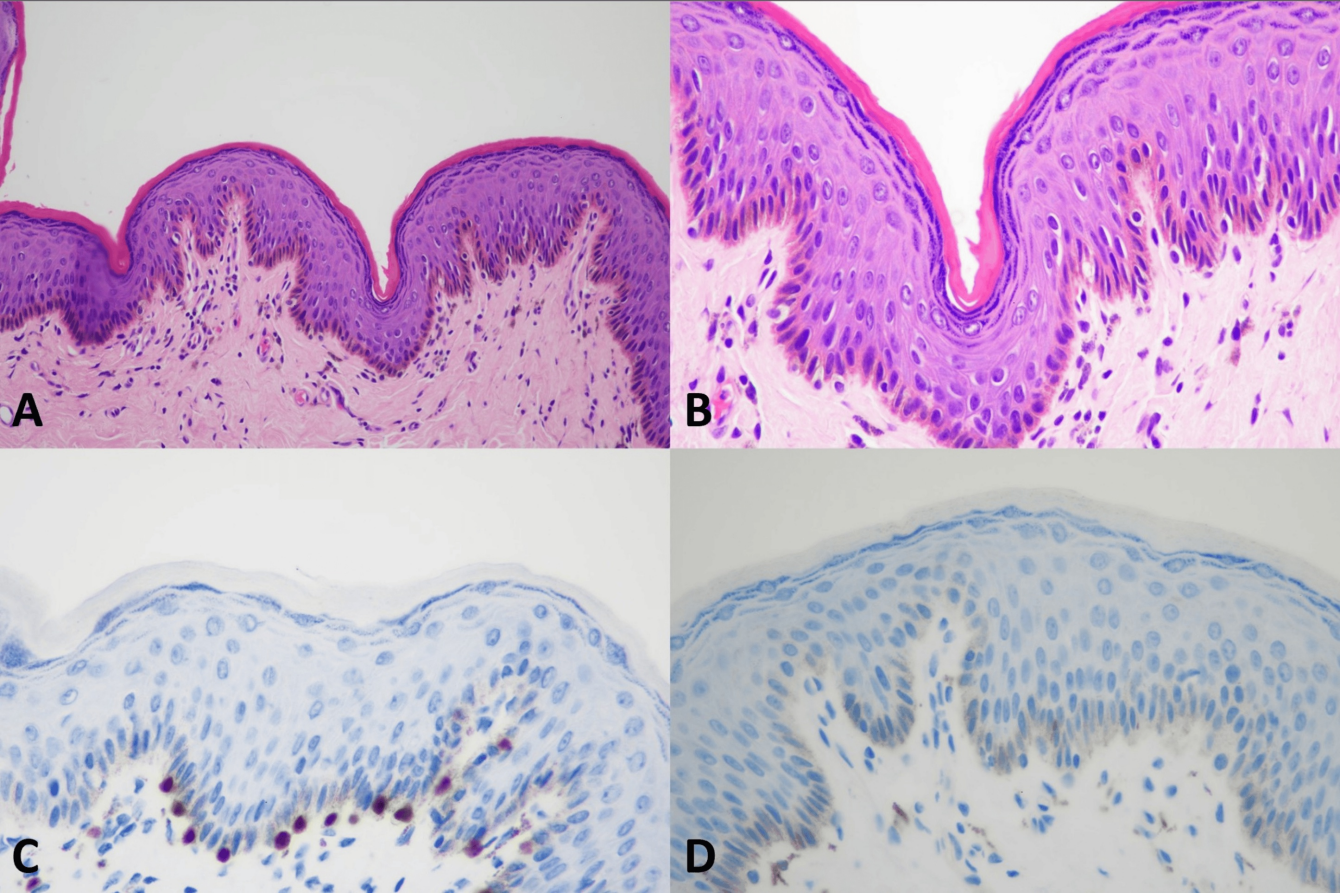
Histopathology of genital lentigine (A) Genital lentigine (original magnification ×100), (B) Genital lentigine (original magnification ×200), (C) Positive nuclear staining of SOX10 (original magnification ×200), (D) Negative nuclear staining of PRAME (original magnification ×200)

**Figure 8:**
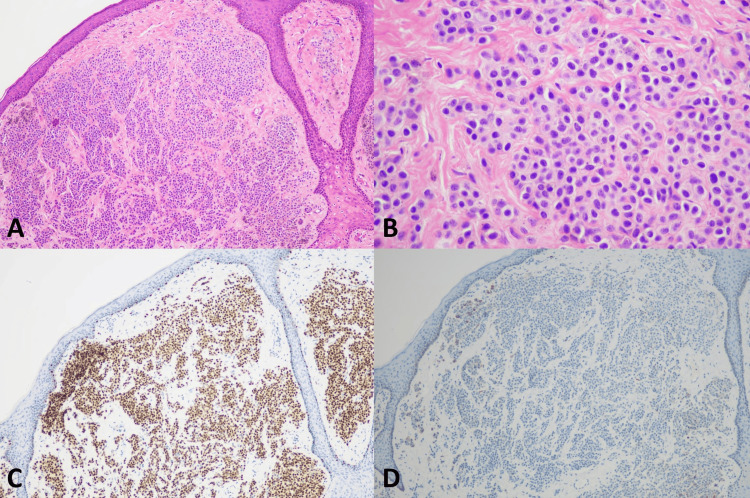
Histopathology of genital melanocytic nevus (A) Genital melanocytic nevus (original magnification ×100), (B) Bland-looking melanocytes (original magnification ×200), (C) Positive nuclear staining of SOX10 (original magnification ×100), (D) Negative nuclear staining of PRAME (original magnification ×100)

SOX10 IHC studies (Table 3) showed positive nuclear staining in all 9 (100%) cases of VM, all 9 (100%) melanoma in situ, and all 13 (100%) benign melanocytic lesions. All the VM cases had diffusely positive (> 75%) nuclear staining and showed strongly positive nuclear staining in 6 of 9 (66.7%) and moderately positive nuclear staining in 3 out of 9 (33.3%). Examples of SOX10 positive in the VM were present in Figures 1-5. All 9 (100%) melanoma in situ lesions also had diffusely strong positive (> 75%) nuclear staining (Figure 6). Benign melanocytic lesions, including GL and melanocytic nevus, showed diffuse (> 75%) and strongly positive nuclear staining in 12 of 13 (92.3%) cases (Figure 7-8).

PRAME IHC studies (Table 3) showed positive stains in 7 out of 9 (77.8%) melanoma cases. Among those with positive PRAME immunostaining, 6 (66.7%) had strongly positive nuclear staining, and 1 (11.11%) had moderately positive nuclear staining. Seven (77.8%) melanomas showed diffusely positive (> 75%) nuclear staining of the neoplastic cells. The examples of melanomas with PRAME positivity were shown in Figures 1-4, whereas a melanoma with PRAME negativity was present in Figure 5. All 9 (100%) melanoma in situ lesions were diffusely positive for PRAME (> 75%). Of them, 5 (55.6%) had moderately positive nuclear staining, and 4 (44.4%) had strongly positive nuclear staining (Figure 6). All 13 (100%) benign melanocytic lesions, which included GL and melanocytic nevi, were negative for PRAME immunostaining (Figure 7-8).

**Table 3:** Immunohistochemical stain results. The statistical test was performed using either the chi-square test or Fisher’s exact test. ^*^ Indicates statistically significant difference (p < 0.05) between SOX10 and PRAME Abbreviations: n, number; χ^2^, chi-square value

		Melanoma (n = 9)				Melanoma in situ (n = 9)				Benign melanocytic lesions (n = 13)			
		SOX10	PRAME	Statistic	p-value	SOX10	PRAME	Statistic	p-value	SOX10	PRAME	Statistic	p-value
Result, n (%)	Positive	9 (100)	7 (77.8)	Chi-square test χ^2^ = 2.25	0.137	9 (100)	9 (100)	Fisher’s exact test	1.000	13 (100)	0 (0)	Chi-square test χ^2^ = 26	< 0.001^*^
	Negative	0 (0)	2 (22.2)			0 (0)	0 (0)			0 (0)	13 (100)		
Strength of positive immunostaining, n (%)	Weak to moderate	3 (33.3)	1 (11.1)	Chi-square test χ^2^ = 0.76	0.383	9 (100)	5 (55.6)	Chi-square test χ^2^ = 5.14	0.023^*^	1 (7.7)	0 (0)	Fisher’s exact test	1.000
	Strong	6 (66.7)	6 (66.7)			0 (0)	4 (44.4)			12 (92.3)	0 (0)		

Regarding the diagnosis of melanoma, SOX10 immunostaining showed sensitivity and specificity of 100% and 0%, respectively, while PRAME immunostaining had sensitivity and specificity of 88. 9% and 100%, respectively. Sensitivity, specificity, positive predictive value, negative predictive value, positive likelihood ratio, negative likelihood ratio, and accuracy of SOX10 and PRAME were reported in Table 4.

**Table 4:** Sensitivity, specificity, positive predictive value (PPV), negative predictive value (NPV), positive likelihood ratio (PLR), negative likelihood ratio (NLR) and accuracy of SOX10 and PRAME immunostaining for diagnosis of melanoma Abbreviation: CI: Confidence interval

	SOX10	PRAME
Sensitivity, % (95% CI)	100 (81.5–100)	88.9 (65.3–98.6)
Specificity, % (95% CI)	0 (0–24.7)	100 (75.3–100)
PPV, % (95% CI)	58.1 (39.1–5.5)	100 (79.4–100)
NPV, % (95% CI)	-	86.7 (63.8–96)
PLR, ratio (95% CI)	1.0 (1.0–1.0)	-
NLR, ratio (95% CI)	-	0.1 (0.03–0.4)
Accuracy, % (95% CI)	58.1 (39.1–75.5)	93.6 (78.6–99.2)

## Discussion

The most prevalent symptoms of VM observed in our study were mass and bleeding, which aligned with previous research [2-5]. Histopathologically, one VM case exhibited a small blue round cell feature, and one VM displayed a pseudoglandular feature. These are rare variants of melanoma that require differentiation from other malignant neoplasms. The small blue round cell feature increases the differential diagnosis with lymphomas, retinoblastoma, hepatoblastoma, neuroblastoma, Wilms’ tumor, Ewing sarcoma/PNET, synovial sarcoma, desmoplastic small round cell tumor, osteosarcoma, and mesenchymal chondrosarcoma, respectively [22]. Pseudoglandular features led to differential diagnosis with adenocarcinomas. Immunohistochemistry is typically employed in this situation. In addition to HMB-45, Melan-A, S100, and tyrosinase, combined SOX10 and PRAME is an adjunct panel of immunohistochemistry for the diagnosis of VM in patients presenting with an unusual feature, based on the sensitivity and specificity of our study.

Immunostaining for melanocytic lesions usually involves S100, Melan A, tyrosinase, and HMB 45 [23]. Our study showed that SOX10 immunostaining was positive in all cases of VM, GL, and melanocytic nevi. PRAME, which is claimed to be more specific for melanoma in most of the studies, was positive in the majority of VM cases and negative for all benign melanocytic lesions in our study. Both SOX10 and PRAME are remarkably easier to interpret due to their nuclear staining rather than cytoplasmic staining in other stains (HMB-45, Melan A), especially in junctional melanocytic lesions. SOX10 has high sensitivity for both benign melanocytic lesions and VM. PRAME immunostaining has been shown to be specific for VM. In other studies, PRAME had a sensitivity of 88.89% and a specificity of 100% and performed well with other combinations of stains such as HMB-45, Melan-A, and Ki-67 [24, 25]. Based on our results and previous studies, PRAME should be able to diagnose VM with high specificity [17, 20, 26].

GL has been found to be related to VM. GL was presented in five cases of VM in our study. In addition to GL, our study showed that all VM cases showed adjacent melanoma in situ, raising the possibility of development from GL to melanoma in situ to VM. However, there was no direct study evaluating the association between genital melanosis and VM. In addition, our study demonstrated that all melanoma in situ is positive for PRAME, while all GL are negative for PRAME. The evaluation of resection margins can be challenging in VM with melanoma in situ, accompanied by GL, which presents as junctional lentiginous proliferation resembling melanoma in situ. Conventional melanocytic immunostaining panels (HMB-45, Melan A, SOX10) are not sufficient to differentiate them, because both benign and malignant melanocytic proliferations can show the same positive staining. A potential solution to this problem is to use PRAME immunostaining specificity, which can distinguish between GL and melanoma in situ [20, 27]. In this study, we compared the SOX-10 and PRAME immunohistochemistry only lesion of VM, melanoma in situ and benign melanocytic lesions without margin evaluation. Further investigation of PRAME and SOX10 to evaluate the margin resection of VM is recommended in another study design.

Because melanoma arising in the vulva is a relatively rare disease, the major limitation of our study was the small sample size. In addition, benign melanocytic lesions, including common melanocytic nevi and congenital melanocytic nevi, in our study can mostly be diagnosed based on histopathology. We had five GL cases in our study, which mainly involved differential diagnosis with melanoma in situ and only one case of special site nevus, which is the principal differential diagnosis with VM. Even though we found statistically significant negative immunoreactivity of PRAME in benign vulvar melanocytic lesions, most VMs (77.8%) revealed a positive PRAME stain. Further investigations for melanocytic lesions in this region still need more studies and a greater number of lesions.

## Conclusions

VM is a rare malignancy of the vulva and is mostly presented with a mass and bleeding. The most common histologic features were epithelioid patterns. Rare histomorphology could be found and lead to additional melanocytic immunohistochemistry studies for a definite diagnosis. SOX10 and PRAME are nuclear staining markers that can be used for diagnostic purposes and adjunct markers for the diagnosis of VM and benign melanocytic lesions. PRAME immunostaining shows high specificity, as there is no positive staining for melanocytic nevi or GL, but positive staining for melanoma and melanoma in situ. However, caution for application, PRAME-negative VM can be found, and this study had a small number of VM or benign melanocytic nevi.

## References

[1] Sturgeon SR, Brinton LA, Devesa SS, Kurman RJ (1992). In situ and invasive vulvar cancer incidence trends (1973 to 1987). Am J Obstet Gynecol.

[2] Sinasac SE, Petrella TM, Rouzbahman M, Sade S, Ghazarian D, Vicus D (2019). Melanoma of the vulva and vagina: Surgical management and outcomes based on a clinicopathologic review of 68 cases. J Obstet Gynaecol Can.

[3] Skovsted S, Nielsen K, Blaakaer J (2017). Melanomas of the vulva and vagina. Dan Med J.

[4] Wohlmuth C, Wohlmuth-Wieser I, May T, Vicus D, Gien LT, Laframboise S (2020). Malignant melanoma of the vulva and vagina: A US population-based study of 1863 patients. Am J Clin Dermatol.

[5] Ragnarsson-Olding B, Johansson H, Rutqvist LE, Ringborg U (1993). Malignant melanoma of the vulva and vagina: Trends in incidence, age distribution, and long-term survival among 245 consecutive cases in Sweden 1960-1984. Cancer.

[6] Ragnarsson-Olding BK, Kanter-Lewensohn LR, Lagerlof B, Nilsson BR, Ringborg UK (1999). Malignant melanoma of the vulva in a nationwide, 25-year study of 219 Swedish females: Clinical observations and histopathologic features. Cancer.

[7] Nakhleh RE, Wick MR, Rocamora A, Swanson PE, Dehner LP (1990). Morphologic diversity in malignant melanomas. Am J Clin Pathol.

[8] Janco JM, Markovic SN, Weaver AL, Cliby WA (2013). Vulvar and vaginal melanoma: case series and review of current management options including neoadjuvant chemotherapy. Gynecol Oncol.

[9] Ragnarsson-Olding BK, Nilsson BR, Kanter-Lewensohn LR, Lagerlof B, Ringborg UK (1999). Malignant melanoma of the vulva in a nationwide, 25-year study of 219 Swedish females: Predictors of survival. Cancer.

[10] Hahn HM, Lee KG, Choi W, Cheong SH, Myung KB, Hahn HJ (2019). An updated review of mucosal melanoma: Survival meta-analysis. Mol Clin Oncol.

[11] Verschraegen CF, Benjapibal M, Supakarapongkul W (2001). Vulvar melanoma at the M. D. Anderson Cancer Center: 25 years later. Int J Gynecol Cancer.

[12] Shah KN (2010). The risk of melanoma and neurocutaneous melanosis associated with congenital melanocytic nevi. Semin Cutan Med Surg.

[13] Yarak S, Michalany NS, Heinke T (2012). Clinical and histopathological characteristics of genital melanocytic nevi: A report of 109 cases and a review of the literature. J Clin Exp Dermatol Res.

[14] Mohamed A, Gonzalez RS, Lawson D, Wang J, Cohen C (2013). SOX10 expression in malignant melanoma, carcinoma, and normal tissues. Appl Immunohistochem Mol Morphol.

[15] Agnarsdóttir M, Sooman L, Bolander A (2010). SOX10 expression in superficial spreading and nodular malignant melanomas. Melanoma Res.

[16] Ordóñez NG (2013). Value of SOX10 immunostaining in tumor diagnosis. Adv Anat Pathol.

[17] Lezcano C, Jungbluth AA, Busam KJ (2021). PRAME immunohistochemistry as an ancillary test for the assessment of melanocytic lesions. Surg Pathol Clin.

[18] Lezcano C, Jungbluth AA, Nehal KS, Hollmann TJ, Busam KJ (2018). PRAME expression in melanocytic tumors. Am J Surg Pathol.

[19] Lezcano C, Jungbluth AA, Busam KJ (2020). Comparison of immunohistochemistry for PRAME with cytogenetic test results in the evaluation of challenging melanocytic tumors. Am J Surg Pathol.

[20] Fattori A, de la Fouchardière A, Cribier B, Mitcov M (2022). Preferentially expressed Antigen in MElanoma immunohistochemistry as an adjunct for evaluating ambiguous melanocytic proliferation. Hum Pathol.

[21] Bastian BC, Lazar AJ, Scolyer RA (2020). Melanocytic neoplasms. Skin Tumours.

[22] Sharma S, Kamala R, Nair D (2017). Round cell tumors: Classification and immunohistochemistry. Indian J Med Paediatr Oncol.

[23] Ohsie SJ, Sarantopoulos GP, Cochran AJ, Binder SW (2008). Immunohistochemical characteristics of melanoma. J Cutan Pathol.

[24] Kunc M, Żemierowska N, Skowronek F, Biernat W (2023). Diagnostic test accuracy meta-analysis of PRAME in distinguishing primary cutaneous melanomas from benign melanocytic lesions. Histopathology.

[25] Grillini M, Ricci C, Pino V, Pedrini S, Fiorentino M, Corti B (2022). HMB45/PRAME, a novel double staining for the diagnosis of melanocytic neoplasms: technical aspects, results, and comparison with other commercially available staining (PRAME and Melan A/PRAME). Appl Immunohistochem Mol Morphol.

[26] Hu J, Cai X, Lv JJ (2022). Preferentially expressed antigen in melanoma immunohistochemistry as an adjunct for differential diagnosis in acral lentiginous melanoma and acral nevi. Hum Pathol.

[27] Gradecki SE, Valdes-Rodriguez R, Wick MR, Gru AA (2021). PRAME immunohistochemistry as an adjunct for diagnosis and histological margin assessment in lentigo maligna. Histopathology.

